# Effects of contaminants of emerging concern on *Megaselia scalaris* (Lowe, Diptera: Phoridae) and its microbial community

**DOI:** 10.1038/s41598-017-08683-7

**Published:** 2017-08-15

**Authors:** Marcus J. Pennington, Jason A. Rothman, Michael B. Jones, Quinn S. McFrederick, Jay Gan, John T. Trumble

**Affiliations:** 1 Department of Entomology, University of California, Riverside, CA 92521 USA; 2 Graduate Program in Environmental Toxicology, University of California, Riverside, CA 92521 USA; 3 Department of Environmental Chemistry, University of California, Riverside, CA 92521 USA; 4 Graduate Program in Microbiology, University of California, Riverside, CA 92521 USA

## Abstract

Drought, rising temperatures, and expanding human populations are increasing water demands. Many countries are extending potable water supplies by irrigating crops with wastewater. Unfortunately, wastewater contains biologically active, long-lived pharmaceuticals, even after treatment. Run-off from farms and wastewater treatment plant overflows contribute high concentrations of pharmaceuticals to the environment. This study assessed the effects of common pharmaceuticals on a cosmopolitan saprophagous insect, *Megaselia scalaris* (Diptera: Phoridae). Larvae were reared on artificial diets spiked with contaminants of emerging concern (CECs) at environmentally relevant concentrations. Female flies showed no oviposition preference for treated or untreated diets. Larvae exposed to caffeine in diets showed increased mortality, and larvae fed antibiotics and hormones showed signs of slowed development, especially in females. The normal sex ratio observed in *M*. *scalaris* from control diets was affected by exposure to caffeine and pharmaceutical mixture treatments. There was an overall effect of treatment on the flies’ microbial communities; notably, caffeine fed insects displayed higher microbial variability. Eight bacterial families accounted for approximately 95% of the total microbes in diet and insects. Our results suggest that CECs at environmentally relevant concentrations can affect the biology and microbial communities of an insect of ecological and medical importance.

## Introduction

Pharmaceuticals have been increasingly prescribed for the past 30 years, and prescription rates have almost tripled in the past 14 years^1, 2^. In food-producing animals alone, there were 9.1 million kg of medically important antibiotics (antibiotics used in both humans and animals) used in 2013. Of those 9.1 million kg used, 73.6% was used for the purpose of increasing production of the animals, and this use continues to increase^3^. Many antibiotics and other common Contaminants of Emerging Concern (CECs) (acetaminophen, mental stimulants, heartburn medications, allergy, and bacterial infection treatments), are excreted by both humans and animals with little change in their chemical structure^4^. It is no surprise pharmaceuticals have been appearing in wastewater, and in some cases tap water, over the past few years^5, 6^.

Standard wastewater treatment facilities are ill equipped to remove pharmaceuticals^7, 8^. Many pharmaceuticals are released during heavy storms in the untreated wastewater, due to overflow, which then flows directly to the environment^9^. These pharmaceuticals are now found at biologically active concentrations in surface waters around the world^10–14^. In addition to runoff, there is an increasing effort to use reclaimed wastewater in drought affected areas, such as Southern California^15, 16^. In agriculture/livestock operations, pharmaceuticals are also found in manure that is then used as fertilizer, effectively compounding the pharmaceutical concentrations^10, 17, 18^. Current research shows these chemicals tend to be both long lived in soil and detrimental to soil microbes^11, 19–22^.

Recent studies on the effects of pharmaceuticals on aquatic insects show that at environmentally relevant concentrations they can alter development of the mosquito *Culex quinquefaciatus*, its susceptibility to a common larvicide, and its larval microbial communities^23, 24^. Watts *et al*.^25^ showed alterations and deformities in the midge *Chironomus riparius* after treatment with a common birth control agent, 17α-ethinylestradiol, and a common plasticizer, Bisphenol-A. Interestingly, many chemicals used by humans, which are not intended for use on microbial communities, have been shown to affect microbes. For example, caffeine, a common mental stimulant, alters biofilm respiration, and an antihistamine, diphenhydramine, has been demonstrated to modify the microbial community and respiration of lake biofilms^26^. Because of unexpected pharmaceutical effects, it is relatively difficult to predict what will occur in model organisms. This problem is exacerbated by a lack of information regarding pharmaceuticals’ effects on terrestrial insects: no available publications report the effects on any terrestrial insects’ microbial community.

Arthropods, such as insects and crustaceans, rely on hormones to grow, develop, mate and even produce pigmentation^27–29^. However, many pharmaceuticals, especially hormones, resemble chemicals that these organisms rely on for growth and development. These pharmaceuticals then bind to receptors and either over-express or suppress their counterparts’ natural function. This has been reported in birds, reptiles, and arthropods where endocrine disruption occurs, primary and secondary sexual characteristics are modified, and courtship behaviors change^27, 30–34^. While most arthropod hormones do not closely match those of mammals, their molting hormone (ecdysone), is very similar to 17β-estradiol (the mammalian female sex hormone). In crustaceans, mammalian hormones have been known to cause both increased molting events and inhibition of chitobiase, the enzyme responsible for digestion of the cuticle during insect molting^35, 36^. In insects, 17α-ethinylestradiol, a common synthetic birth control hormone, has been shown to alter molting and lead to deformities of *C*. *riparius*. Also, Bisphenol-A, a common plasticizer, can bind and activate estrogen receptors in humans, and the ecdysone-binding protein in insects^25, 37^. In addition to these effects, pharmaceuticals have been shown to cause effects to insects over multiple generations^38^.

*Megaselia scalaris* (Lowe, Diptera: Phoridae) is a common saprophagous pest. They are known to infect living humans (myiasis), provide important ecological roles as detritivores, and because they often feed on human corpses are commonly used in forensic entomology to determine time of death^39, 40^. This species will generally feed on a variety of decomposing plant and animal tissues, and acts as a vector of pathogens^39, 41^. These insects are both fecund and hardy because females can lay over 650 eggs in 16 days and are tolerant of heavy metals^42, 43^. The white, roughly football-shaped eggs, hatch after approximately 24 hours into white translucent larvae. When they have matured to third instar (life-stage) they pupariate^39, 40^. Their detritivorous larval life history exposes them to a wide diversity of microorganisms that may act as pathogens, commensals, and symbionts. There is currently no record of how *M*. *scalaris* acquires their microbiota or if any symbionts are required. However, it stands to reason that they, like so many other insects, would rely on microbial symbionts^44, 45^. There are many ways insects acquire symbionts: from their diet, the environment, their social network, or vertical transmittance (maternally inherited)^46–49^.

Currently there is little to no information regarding pharmaceutical effects at the concentrations found in reclaimed water on the growth or microbial community composition of any terrestrial detritivore. These detritvores become exposed to contaminants after the CECs enter surface waters, soil, and plants from overflow and wastewater reuse. There are studies involving antibiotics at high doses to determine necessity of microbiota in several insects, but these have not tested relevant concentrations found in reclaimed water or joint effects of other pharmaceuticals, which often coexist with antibiotics^45, 50^. To assess potential effects of common pharmaceuticals, we used a series of bioassays to determine the possibility of individual and joint contamination on development, mortality and population sex ratios of *M*. *scalaris*. Any effects would have potentially important implications from medical, ecological, and forensic perspectives. Also, as there is currently no information on *M*. *scalaris*’ microbial community, information generated from this study could serve as novel information into the role possible symbionts play in *M*. *scalaris* development.

## Results

### Oviposition Choice, Mortality, Days to Pupariation, and Sex Ratio Differences

**Figure 1:**
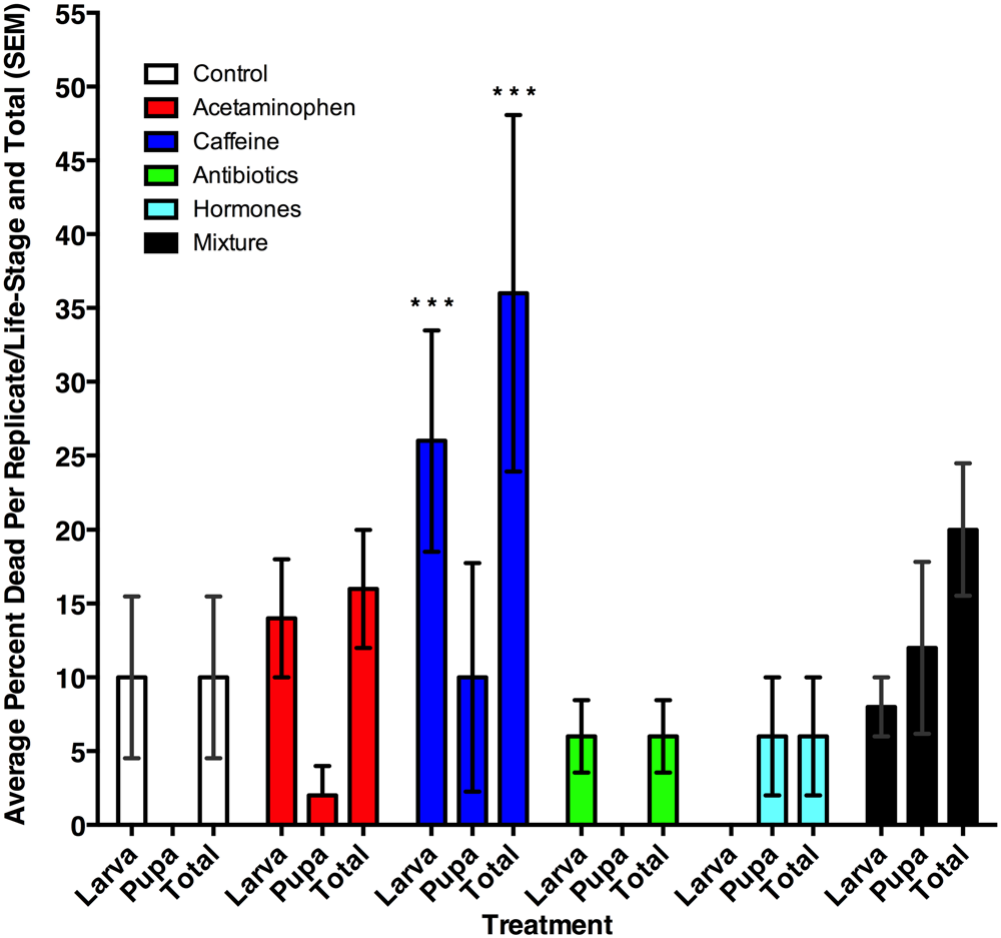
Average (SEM) mortality of larvae, pupae, and total insects for each treatment group. ***Denotes significant difference (α = 0.05) relative to the control.

These insects did not respond to these contaminants because pharmaceuticals in reclaimed water did not affect oviposition preferences (χ^2^ = 3.66, df = 5, p = 0.60). Mortality was increased (χ^2^ = 23.21, df = 5, p < 0.001) when *M*. *scalaris* were fed diets containing caffeine (p < 0.01) (Fig. 1). Increased mortality was evident both in the larval life-stage (χ^2^ = 22.81, df = 5, p < 0.001) and the pupal stage (χ^2^ = 17.41, df = 5, p < 0.01). The largest increase in mortality was in larval-stage caffeine treatments (p < 0.05).

**Figure 2:**
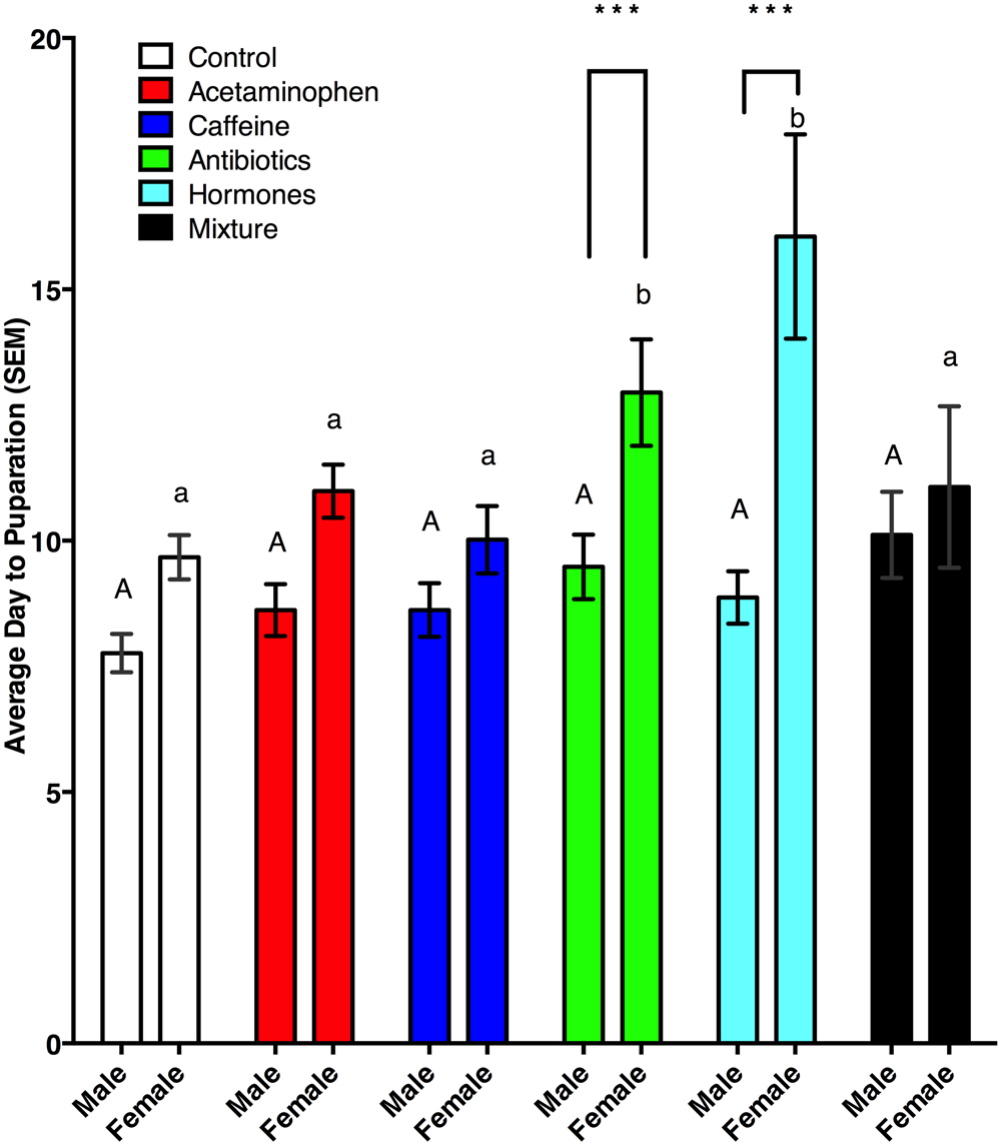
Average day to pupariation of male and female *Megaselia scalaris* by treatment. Upper case letters denote significant differences in days to pupariation (DTP) from male control. Lower case letters denote significant differences in DTP from female control. ***Denotes an overall day to pupariation difference from controls.

**Figure 3:**
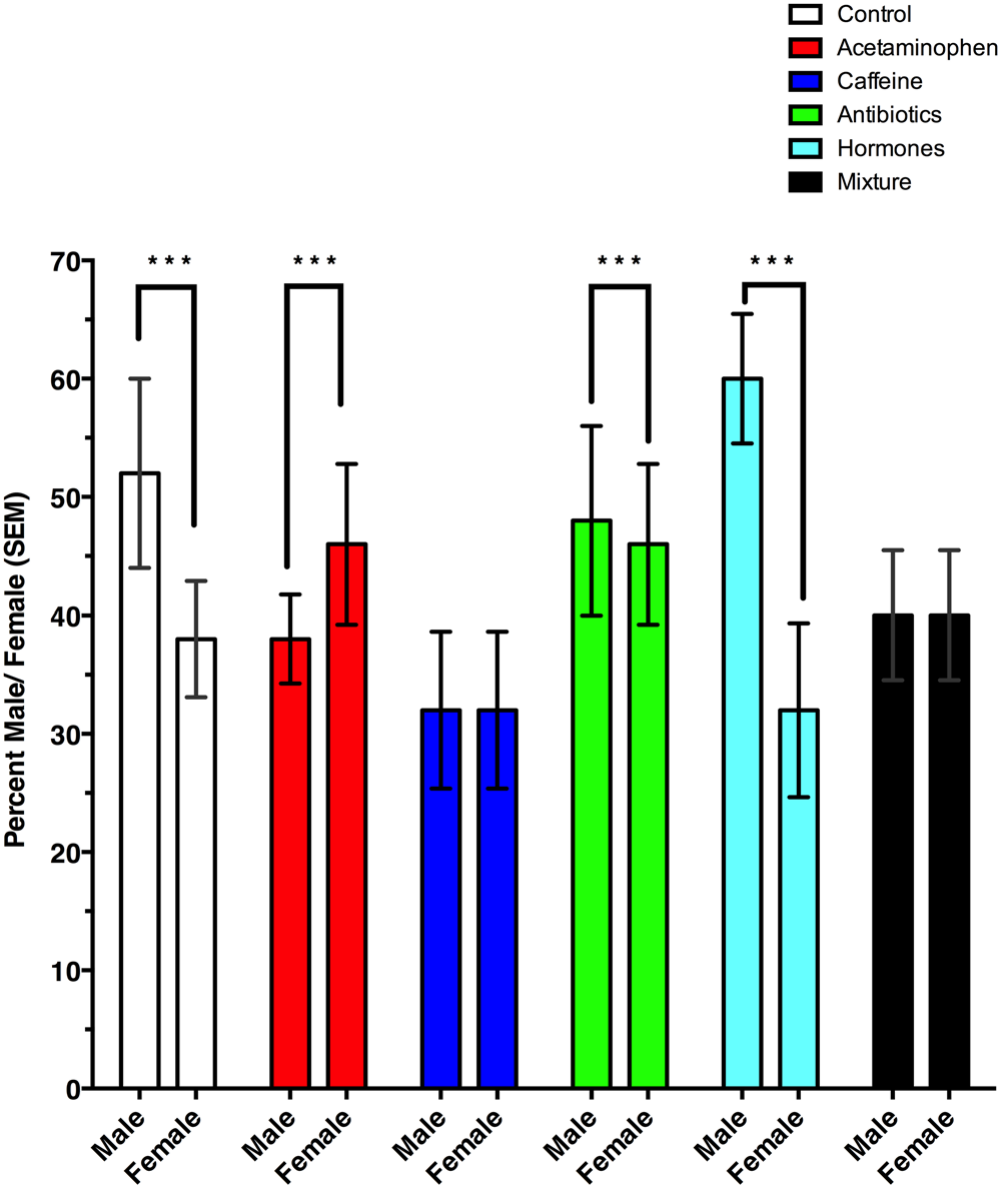
Male: female ratios of *Megaselia scalaris* fed diets contaminated with common pharmaceuticals found in reclaimed water. ***Denotes a significant difference in sex ratio with respect to treatment.

There was an increased time to pupariation (χ^2^ = 24.71, df = 5, p < 0.001) for *M*. *scalaris* fed antibiotic (p < 0.01) or hormone (p < 0.001) containing diets (Fig. 2). While there were no overall differences of sex ratio (χ^2^ = 4.54, df = 5, p = 0.48), sex did have an effect on pupariation time (χ^2^ = 52.59, df = 1, p < 0.001), and there was an interaction of treatment and sex (χ^2^ = 26.88, df = 5, p < 0.001), which was most evident in the mixture treatment (p < 0.01). Within individual treatments, however, there were sex ratio differences (Fig. 3) for *M*. *scalaris* exposed to diets in the control (p < 0.05), acetaminophen (p < 0.05), antibiotics (p < 0.001), and hormone (p < 0.001) treatments. Interestingly, there were no sex ratio differences in the caffeine (p = 0.15) and mixture (p = 0.88) treatments. Comparing the time to pupariation of opposite sexes (χ^2^ = 44.25, df = 5, p < 0.001), male times to pupariation in treatments were not different (χ^2^ = 7.34, df = 5, p = 0.20) than the controls. However, female (χ^2^ = 44.25, df = 5, p < 0.001) development in the antibiotic (p < 0.01) and hormone (p < 0.001) treated diets took significantly longer to pupariate than the control females (Fig. 2).

### Bacterial Community Analysis

**Figure 4:**
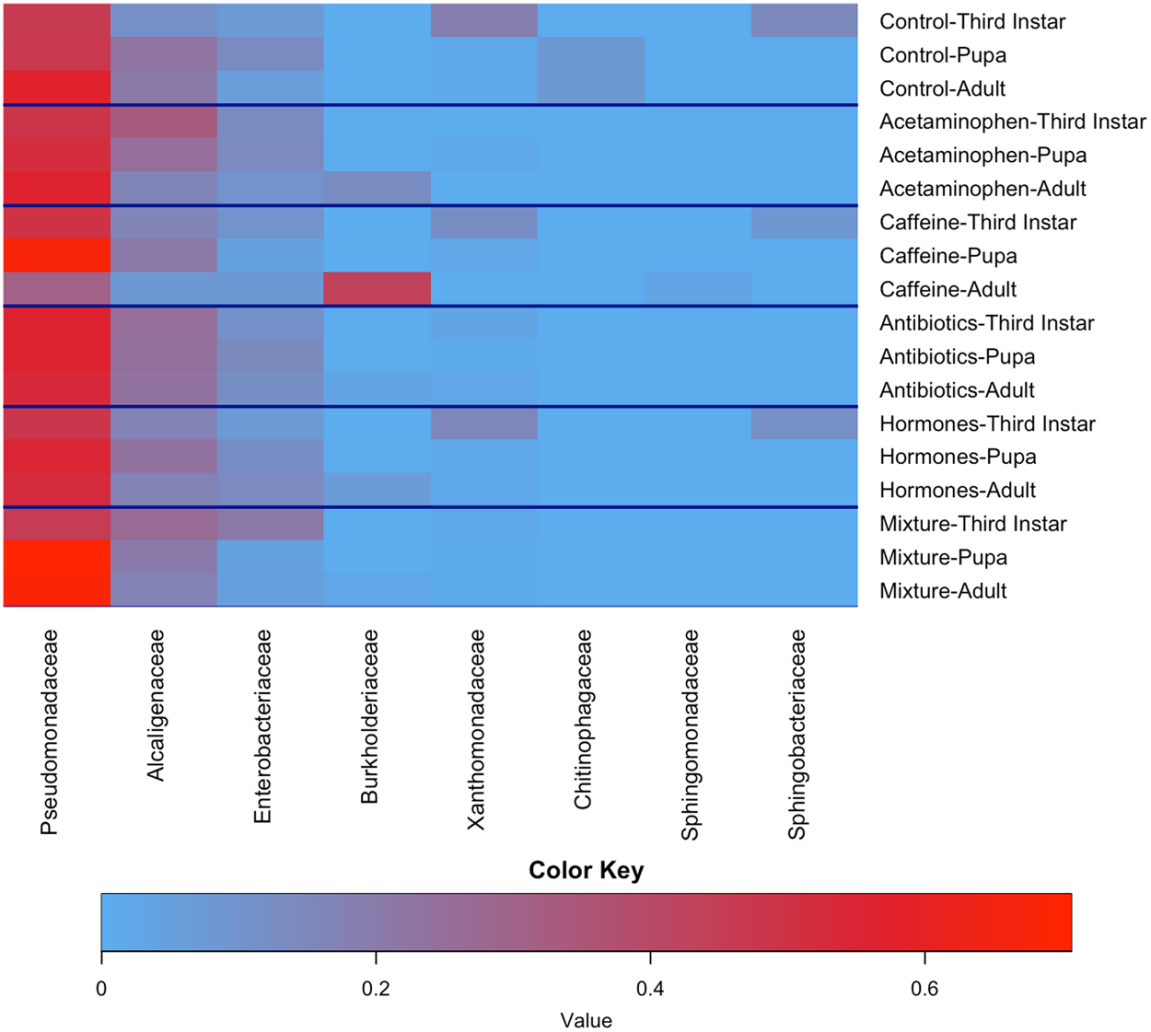
Heatmap of the most abundant bacterial families (each accounting for at least 2.5% of the total OTUs) by average OTUs of treatment life-stage pairing Increased red coloration is an increase in indicative of increased proportional abundance.

There were 752,855 total raw reads, with an average of 10,456 reads per sample, and a total of 772 distinct operational taxonomic units (OTUs), DNA sequences which are at least 97% identical, after removing OTUs identified as mitochondria, chloroplast, and obvious contaminant DNA through BLAST analysis. Overall, there was an effect of treatment (Adonis PERMANOVA: F = 1.92; df = 5, 44; p < 0.05) on the bacterial community of *M*. *scalaris*. Based on adjusted p-values (BH False Discovery Rate), a majority of differences in the treatments occurred between third instars and adults (Table 1). There were 30 different OTUs responsible for these differences and of those 28 were different in the third instar/adult stage comparisons. There were eight bacterial families that accounted for at least 2.5% (by proportional abundance of OTUs) of the total bacterial families found in at least one sample (Fig. 4 and Table 2) and, collectively, they account for at least 94% of the total microbial community found in all life-stages. Six of the eight families in Fig. 4 showed differences between third instars and adults (Table 1). Only *Burkholderiaceae* did not show a difference between third instar and pupal stages. *Xanthomonadaceae* and *Sphingomonadaceae* were the only families on Fig. 4 not also in Table 1. According to a non-metric multidimensional scaling (NMDS) plot (Supp. Fig. 2), the least dissimilarities were observed among (1) pupae and diets from hormone treatments, (2) the adults, pupae, and diets, from mixture treatments and (3) the individuals exposed to antibiotics.

**Table 1:** Bacterial families and genera in each treatment that are significantly different in at least one life-stage pairing.

Treatment	Phylum	Family	Genus	Species	Third Instar-Pupa	Third Instar- Adult	Pupa-Adult
Control	Actinobacteria	*Corynebacteriaceae*	*Corynebacterium*	*sp*.	*		
	Bacteroidetes	*Chitinophagaceae*	*Sediminibacterium*	*sp*.	*	*	
	Proteobacteria	*Alcaligenaceae*	*Achromobacter*	*sp*.	*	*	
		*Caulobacteraceae*	*Caulobacter*	*sp*.	*	*	
		*Enterobacteriaceae*	*Klebsiella*	*sp*.	*	*	
			*Enterobacter*	*sp*.	*		
		*Erwiniaceae*	*Erwinia*	*sp*.	*	*	
		*Hyphomicrobiaceae*	*Pedomicrobium*	*sp*.		*	
		*Methylobacteriaceae*	*Methylobacterium*	*sp*.	*	*	
		NA	NA	*sp*.	*	*	
		*Pseudomonadaceae*	*Pseudomonas*	*veronii*	*	*	
				*sp*.	*	*	
				*sp*.	*	*	
		*Sinobacteraceae*	*Steroidobacter*	*sp*.		*	
Acetaminophen	Proteobacteria	*Pseudomonadaceae*	*Pseudomonas*	*sp*.		*	
Caffeine	Actinobacteria	*Cornebacteriacea*	*Corynebacterium*	*sp*.			*
	Proteobacteria	*Bradyrhizobiaceae*	*Afipia*	*sp*.		*	*
		*Burkholderiaceae*	*Burkholderia*	*sp*.		*	*
		*Enterobacteriaceae*	*Enterobacter*	*sp*.	*	*	
		*Erythrobacteraceae*	*Altererythrobacter*	*sp*.		*	*
		*Methylobacteriaceae*	*Methylobacterium*	*sp*.		*	*
				*sp*.		*	*
		*Pseudomonadaceae*	*Pseudomonas*	*sp*.	*	*	
				*sp*.		*	*
Antibiotics	Actinobacteria	*Microbacteriaceae*	*Microbacterium*	*sp*.		*	
		*Micrococcaceae*	*Micrococcus*	*sp*.		*	
	Firmicutes	*Lactobacillaceae*	*Lactobacillus*	*brevis*	*	*	
	Proteobacteria	*Burkholderiaceae*	*Burkholderia*	*sp*.		*	*
Hormones	Actinobacteria	*Mycobacteriaceae*	*Mycobacterium*	*sp*.		*	*
		*Nocardioidaceae*	*Nocardioides*	*sp*.		*	
	Bacteroidetes	*Sphingobacteriaceae*	*Sphingobacterium*	*multivorum*	*	*	
	Proteobacteria	*Alcaligenaceae*	*Achromobacter*	*sp*.	*	*	
		*Enterobacteriaceae*	*Klebsiella*	*sp*.		*	
			*Enterobacter*	*sp*.	*	*	
		*Erwiniaceae*	*Erwinia*	*sp*.	*	*	
		*Methylobacteriaceae*	*Methylobacterium*	*sp*.	*	*	
		*Moraxellaceae*	*Acinetobacter*	*lwoffii*	*	*	
		NA	NA	*sp*.	*	*	
		*Pseudomonadaceae*	*Pseudomonas*	*veronii*	*	*	
				*sp*.	*	*	
				*sp*.	*	*	
		*Rickettsiaceae*	*Rickettsia*	*sp*.		*	*
Mixture	Proteobacteria	*Burkholderiaceae*	*Burkholderia*	*sp*.		*	*
		*Erwiniaceae*	*Erwinia*	*sp*.	*	*	
		*Orbaceae*	*Orbus*	*sp*.		*	

*Denotes adjusted p value of <0.05 in those genera for each life-stage pairing in a treatment.

**Table 2:** Average percentage of bacterial families by insect life-stage.

Bacterial Phylum	Bacterial Family	Avg. Percentage Third Instar	Avg. Percentage Pupa	Avg. Percentage Adult
Proteobacteria	*Alcaligenaceae*	26.41	22.90	17.23
	*Enterobacteriaceae*	13.31	10.64	9.42
	*Pseudomonadaceae*	50.61	58.02	52.26
	*Burkholderiaceae*	0.00	0.02	12.25
	*Xanthomonadaceae*	3.57	1.63	1.21
	*Sphingomonadaceae*	0.16	0.19	0.67
Bacteroidetes	*Chitinophagaceae*	>0.01	1.40	1.49
	*Sphingobacteriaceae*	1.42	0.00	0.02
	Sum Percentages	95.48%	94.80%	94.55%

## Discussion

*Megaselia scalaris*, a common detritivore, has been known to develop on substances as diverse as human wounds and corpses^51, 52^, modeling clay, and emulsion paint^53, 54^. Their ability to grow and mature on these diets, with minimal effect on their survival, and their tolerance to heavy metals^42^ makes any effect of pharmaceuticals at very low doses found in reclaimed water even more surprising. In our study, the females had no preference for untreated diets versus any treated diets. This poses a problem for the insect population, as there was higher larval mortality when developing on a caffeine-contaminated food source. Because females require an additional 24 hours^39^ after emergence in order to be receptive to males, populations exposed to hormones or antibiotics would be adversely affected. If females require an extra six days to emerge and become receptive, there is a reasonable possibility the males would leave the area or perish before mating. In addition, the suitability of decaying food sources tends to be temporary^55^. Collectively, these factors could likely negatively influence population growth. Also, these changes in population growth rate could hinder forensic scientists from determining an accurate time of death if there were long lasting or even moderate concentrations of these pharmaceuticals in the body at death.

Sex ratios of emergent adults were also affected in the caffeine and mixture treatments. The sex ratios found in control treatments in our study are similar to those reported in Benner & Ostermeyer^56^ of a male: female sex ratio at 25 °C of 1.18:1. However, sex ratios from the acetaminophen, caffeine, and mixture treatments differed significantly from the controls. A major difference in sex ratio would change the reproductive capacity of a population. It is unclear why acetaminophen and caffeine would alter sex ratios, however acetaminophen as been recorded to hinder the production of arachadonic acid in mosquitoes, another Dipteran, and it could be playing a similar role here^57^. Ibuprofen, another analgesic and antipyretic has been shown to alter the sex ratio in another mosquito^58^.

Many insects rely on their microbial communities and endosymbionts to grow and develop^59^. However, Adonis, the statistical method used to analyze these data, does not have a post hoc test available that would allow direct pairwise comparisons between treatments. Nonetheless, there are changes in the bacterial community (Fig. 4 and Table 1) based on adjusted p-values evaluating differential abundance. We found significant shifts in the microbial community in the various life stages examined within the control treatments. A similar result has been reported for mosquitoes^24^ and other insects^60, 61^. Not surprisingly, insects that undergo complete metamorphosis and also rely on a different food source as adults would require a different bacterial community; however there is one family, *Pseudomonadaceae*, which appears in all treatments and life-stages. Species in this family are gram-negative Proteobacteria that cannot survive in acidic environments^62^. They are fairly common in insects^63^, and can be responsible for 90 + % of the bacterial community^64^. They are resistant to antibiotics^62^, which potentially explains why they are so prevalent in many of our treatments. *Pseudomonadaceae* is responsible for ~ 50% of the bacteria in all life-stages, followed by *Alcaligenaceae*, *Enterobacteriaceae*, and *Xanthomonadaceae*. *Pseudomonadaceae* and *Enterobacteriaceae* families contain known symbionts in insects^48, 65–67^ and could be filling the same role in *M*. *scalaris*.

When *Pseudomonadaceae* is removed from the heatmap (Supp. Fig. 1), it becomes clear how the next three highly proportional families change with life-stage. *Alcaligenaceae* tends to become more proportionally abundant in pupae and adults than in larvae. Species in the family *Alcaligenaceae* are oxidase- and catalase-positive and utilize a variety of organic and amino acids as carbon sources^68^. *Enterobacteriaceae* has higher proportions in larvae than in adults. Species of *Enterobacteriaceae* are likely to be either symbionts or a pathogen to their hosts^62^. *Enterobacteriaceae* includes *Buchnera*, an important endosymbiont of aphids^69^, and other species that inhabit various insects to provide facultative benefits^48, 70^. *Xanthomonadaceae*, like *Enterobacteriaceae*, is more prominent in larvae than in other life-stages except in acetaminophen, antibiotic, and mixture containing diets, where *Enterobacteriaceae* dominate. Most species in *Xanthomonadaceae* are plant pathogens^62^, and have been known to make use of chitin as a carbon source and utilize insects as vectors^71, 72^. It is possible that some of the bacteria in this family may act as symbionts with insects as they have been found in a variety of insect orders^73–75^.

**Figure 5:**
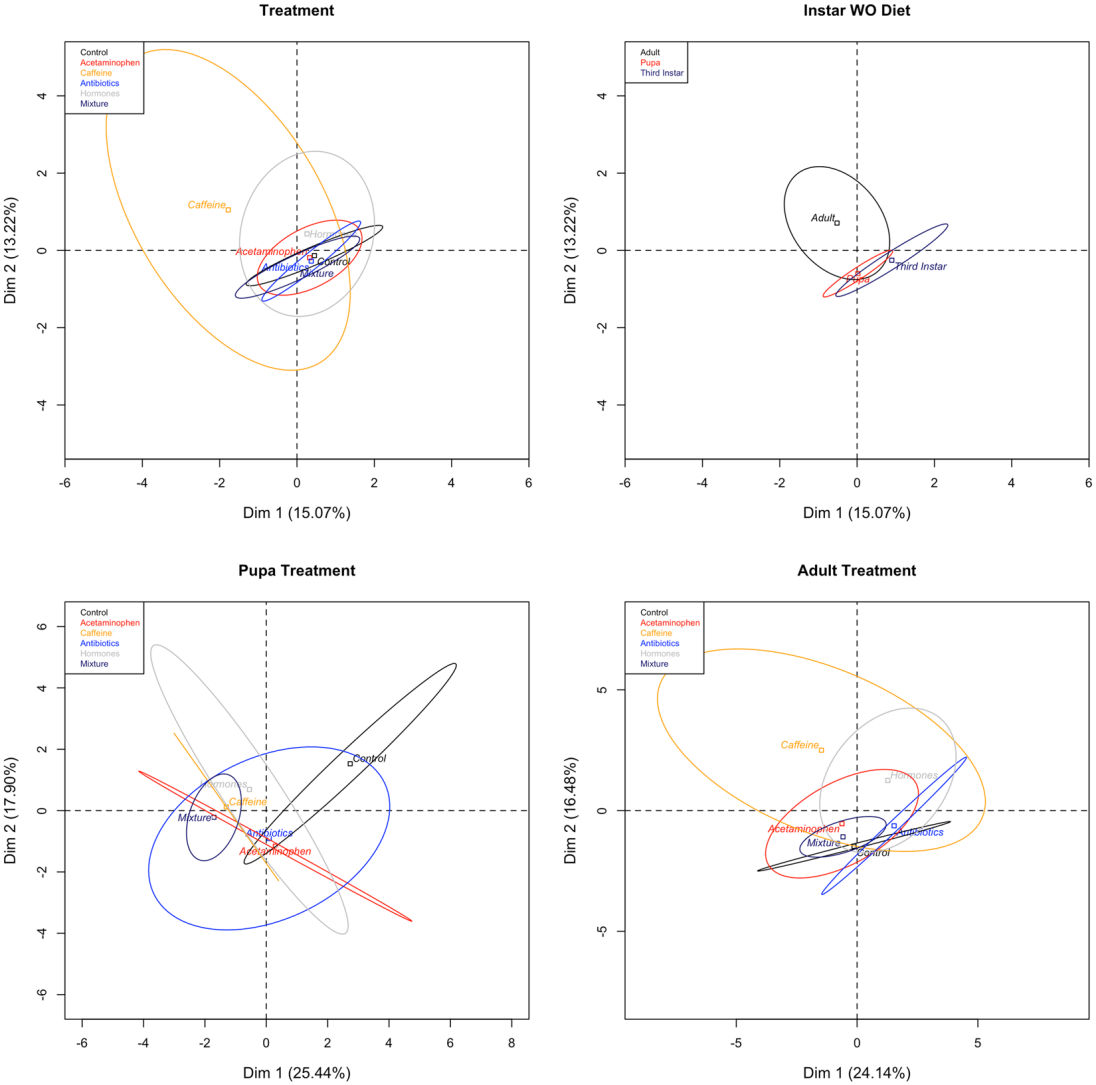
Principal Component Analysis of treatments, life-stage, pupa by treatment, and adult by treatment. Ellipses denote range of individuals around a centroid barycentre.

In the NMDS plot (Supp. Fig. 2) there is distinct clustering in the microbiota by treatment. In individuals exposed to antibiotics in their diets, there is a lack of dissimilarity among their microbial communities. Insects reared on diets containing hormones had less microbial diversity in pupae. Unfortunately, we do not know if this is due to similarity in the microbial communities of third instar individuals as the statistical process of rarefication removed that particular life-stage in larvae exposed to hormones, controls, and caffeine treatments. However, the insects feeding on the mixture treatments show a distinct clustering of microbial groups in the pupal and adult stages, whereas the larval stage contains individuals with more variable microbiota. This is likely due to the early instars being exposed directly to the microbe-laden diet, while the later life-stages are only exposed to a subset of bacteria left after the gut contents were expelled at time of pupariation. The greatest dissimilarity was found in the caffeine-treated adults (Fig. 5 and Supp. Fig. 2). The adult stage, regardless of treatment, also seems to be where the majority of variation in microbiota occurs (Fig. 5).

*Megaselia scalaris* has been suggested as a model organism for bioassays for drugs and pollutants^39^, and our findings support this claim. However, our results also suggest that the presence of even very low concentrations of some pharmaceuticals could affect the forensic estimation of time of death based on emergence patterns of adult *M*. *scalaris*. We also caution that the pharmaceuticals used in this trial were at low concentrations found in wastewater and could be much higher in cadavers, as pharmaceuticals in humans tend to be higher than what is found in the environment^76–79^. Also, due to increases in concentrations caused by water loss (on a weight/weight basis), pharmaceuticals could have higher toxicity in decaying matter. Perhaps most importantly, pharmaceuticals in reclaimed water are having unintended effects on the microbial community of these flies, which could lead to decreased viability of these ecologically useful detritivores.

## Methods and Materials

### Chemicals

Test compounds included: acetaminophen, caffeine, three antibiotics, and four estrogenic steroidal hormones. Six treatments were examined: acetaminophen, caffeine, an antibiotic mixture (lincomycin, oxytetracycline, and ciprofloxacin), a hormone mixture (estrone, 19-norethindrone, 17β- estradiol, and 17α- ethynylestradiol), a mixture of all chemicals (as would be found in overflow or wastewater effluent), and a control, consisting of only distilled water. Distilled water was tested for CECs and found to not contain any. Treatment groups were chosen as representative compounds for pain relievers, mental stimulants, antibiotics commonly used on humans and livestock, hormones normally either produced or prescribed to humans, and as a mixture that would be simple, yet representative of wastewater effluent or reclaimed wastewater. Artificial diets were prepared at room temperature to negate any decomposition of the CECs. Acetaminophen (10 μg/L), caffeine (6 μg/L), estrone (0.112 μg/L), 19-norethindrone (0.872 μg/L), 17β- estradiol (0.2 μg/L), 17α- ethynylestradiol (0.831 μg/L), lincomycin (0.73 μg/L), and oxytetracycline (72.9 μg/L) concentrations were chosen based on the maximum concentrations measured by Kolpin *et al*.^13^. Ciprofloxacin (6,500 μg/L) concentration was chosen from the maximum lake water concentration reported by Mutiyar and Mittal^14^.

The chemicals used were purchased as follows: acetaminophen with a purity of ≥90%; (MP Biomedicals, LLC, Santa Ana, CA); caffeine at laboratory grade purity (Fisher Scientific, Hanover Park, IL); lincomycin, oxytetracycline, and ciprofloxacin with purities of ≥98% (Alfa Aesar, Ward Hill, MA); estrone, 19-norethindrone, 17β- estradiol, and 17α- ethynylestradiol at ≥98% purity (Sigma-Aldrich, St. Louis, MO). Blue formula 4–24^®^ instant *Drosophila* medium, hereafter known as ‘blue diet’, was purchased from Carolina Biological Supply Company (Burlington, NC). Hydrochloric acid (12.1 M) was obtained from Fisher Scientific. Sodium hydroxide was acquired from Sigma-Aldrich (St. Louis, MO) as anhydrous pellets. Stock solutions were prepared by adding powdered chemicals to deionized water. Approximately 5 mL 80% ethanol was added to 250 mL steroidal hormone solutions to facilitate dissolution. Hydrochloric acid (1 M) was added to antibiotic chemical solutions to facilitate dissolution and pH was adjusted using NaOH (1 M) to pH 4.00. Compounds were added to distilled water to the desired concentrations for each treatment and then an equal amount of blue diet flakes was added as described by the manufacturer. In all experiments, preparations and concentrations of treatment groups were prepared as stated previously.

### Insects

A *M*. *scalaris* colony was established in 2015, and the species verified at the Los Angeles Natural History Museum by Ms. Emily Hartop. The colony was maintained on an alfalfa diet as in Mandeville *et al*.^80^. For all experiments, eggs were collected from adults and were stored in an incubator (model 818: Precision Scientific Inc., Buffalo, NY) at 26 °C, approximately 70% RH, and a light: dark cycle of 16:8. In order to standardize the age of larvae in all of the following experiments, eggs were collected by placing 9 cm Petri dishes, containing blue diet, inside the colony for ~12 hr. Petri dishes were wrapped in aluminum foil in a funnel shape to exclude colony larvae. Eggs were then transferred, by microspatula, to bifurcated 9 cm Petri dishes or individual 50 mL centrifuge tubes each containing 25 mL or 2 mL, respectively, of treated or control blue diet. Nine centimeter Petri dishes contained blue diet only on one side of the bifurcation to allow larvae to migrate to the empty side for pupariation. Centrifuge tubes allowed for monitoring of individual larvae, while the Petri dishes were used to rear multiple insects for microbial community analyses.

### Oviposition choice assay

Following blue diet preparation, size 12 cork-borer plugs were taken from each Petri dish. Ten individual 9 cm Petri dish arenas were prepared by placing one plug of each treatment and the control in a circle (6 plugs per dish), with plugs placed equidistantly. Ten male/female pairings were added to each arena and allowed to choose and oviposit for 24 h. Eggs on each plug in each replicate were then counted and recorded.

### Mortality, Days to Pupariation, and Sex differences

Individual eggs were transferred to 50 mL centrifuge tubes by microspatula. There were 10 centrifuge tubes per replicate, and 5 replicates for each treatment (n = 50/n = 300 across all treatments). Inside each centrifuge tube, a strip of filter paper was placed inside to reduce excess moisture and provide a pupariation surface. Individuals were monitored daily for pupariation and adult emergence until all individuals had emerged or died. Adults were then sacrificed at −60 °C, and their sexes were determined based on the structure of genitalia.

### Insect rearing for bacterial analysis

Eggs were transferred, by microspatula, to the blue diet of each 9 cm Petri dish. There were 3 replicates per life stage for each of the 6 treatments (n = 54). Lids contained a size 7 cork-borer hole affixed with 2 layers of fine organza mesh to allow for moisture and gas exchange. Following egg placement, Petri dish lids and bottoms were aligned and secured with parafilm. Petri dishes were monitored daily for development. Six individuals were collected at third instar, pupa, and adult life-stages, triple washed with 200 proof ethanol, and stored in clean 200 proof ethanol at −60 °C until DNA extraction. During the collection of each treatment group and life-stage, blanks in triplicate of DDI H_2_O were used to monitor contamination. Before extraction, triplicate blanks were pooled.

### DNA extractions and Illumina sequencing of whole body Megaselia scalaris bacteria

All DNA extractions and Illumina preparations were performed as in McFrederick and Rehan^46^. Briefly, DNA extractions were performed using a DNeasy Blood and Tissue kit (Qiagen, Valencia, CA). An individual from each life-stage (n = 3), each treatment group (n = 6), and replicate group (n = 3), along with triplicates of the pooled blank (DDI H_2_O), for each treatment group (n = 18), were acquired (n = 72) and placed in individual wells of a 96-well plate provided in the kit. Into each well, we added 180 μL of buffer ATL, a sterile 3.2 mm chrome-steel bead and 100 μL of 0.1 mm glass (Biospec, Bartlesville, OK). A Qiagen tissuelyzer was then used to bead-beat each sample for 6 min at 30 Hz. We then added 20 μL of Proteinase K to each sample and incubated at 57 °C overnight. The standard DNeasy extraction protocol was then followed.

Following extraction, dual-index inline barcoding was used to prepare libraries for sequencing on the Illumina MiSeq. We used primers that included either the forward or reverse Illumina sequencing primer, a unique eight-nucleotide long barcode, and the forward or reverse genomic oligonucleotide as in Kembel *et al*.^81^. For the bacterial 16S rDNA sequences we used the primers 799F-mod3 CMGGATTAGATACCCKGG^82^ and 1115 R AGGGTTGCGCTCGTTG^81^, which have been shown to minimize contamination from plastids.

We used these primers to generate 16S rRNA gene amplicons for Illumina sequencing using PCR. PCRs were performed using 10 μL ultrapure water, 10 μL of 2× Pfusion High-Fidelity DNA polymerase (New England Biolabs, Ipswich, MA), 0.5 μL of each 10 μM primer stock, and 4 μL of DNA. We used a 52 °C annealing temperature, 35 cycles, and negative controls for each reaction. To remove unincorporated primers and dNTPs, we used the Ultraclean PCR clean up kit (MoBio, Carlsbad, CA). We used 1 μL of the clean PCR product as a template for another PCR, using HPLC purified primers to complete the Illumina sequencing construct as in Kembel *et al*.^81^: CAAGCAGAAGACGGCATACGAGATCGGTCTCGGCATTCCTGC, and AATGATACG GCGACCACCGAGATCTACACTCTTTCCCTACACGACG. For the reactions, we used a 58 °C annealing temperature, 35 cycles and negative controls. Once the PCR cycles were finished, we used 18 μL of the PCR product and SequalPrep Normilization plates (ThermoFisher Scientific, Waltham, MA) to normalize the amount of DNA in each sample. We pooled 5 μL of each normalized sample, performed another cleanup, and then used a 2100 Bioanalyzer (Agilent, Santa Clara, CA) to assess our library quality. After quality control, we sequenced the libraries using the MiSeq Reagent kit v3 with 2 × 300 cycles. Raw data are available on the NCBI Sequence Read Archive (SRA) accession number SRP099221.

### Bioinformatics

All genomic information was processed using macQIIME ver. 1.9.1–20150604^83^. We used USEARCH v6.1^84^ to identify and remove chimeric sequences, and SUMACLUST^85^ to cluster OTUs and remove any with less than two reads per sample. We used 97% sequence identity to bin OTUs and choose representative OTUs. We then performed standard alpha and beta diversity analyses in QIIME. To assign taxonomy to OTUs, Greengenes taxonomy^86^ and the RDP Naïve Bayesian Classifier^87^ were utilized, and we also performed BLASTN searches against NCBI’s online Nucleotide Collection (nr/nt) and 16 S ribosomal RNA sequences (Bacteria and Archea) databases (accessed January 17, 2017). Taxonomy was then used to identify any mitochondria or chloroplast OTUs, which were removed from the dataset as in McFrederick & Rehan^46^. We aligned the quality-filtered dataset using the pynast aligner^88^ and the Greengenes database^86^. We then reconstructed the phylogeny of the bacterial OTUs using FASTTREE version 2.1.3^89^. Next we performed weighted and unweighted UniFrac analyses^90^ using the generated phylogeny and OTU tables. Using the generated distance matrices, we performed Adonis^91^ and created PCA^92^ graphs in R version 3.3.1^93^. For alpha diversity, we plotted rarefaction curves in GraphPad Prism version 6.00 software (La Jolla, California), and used gplots^94^ to create a heatmap of the most abundant bacterial families; a 0.025 proportional abundance in at least one sample was used as the cutoff.

### Statistics

All statistical analyses were performed using R (version 3.3.1). Normality was determined using Shapiro-Wilk normality tests. Mortality was determined using a generalized linear model with a binomial family. Differences in days to pupariation were determined using the ‘survival’ and the ‘OIsurv’ packages^95, 96^. In all cases, when data were not considered normal, either a Poisson distribution or a negative binomial generalized linear model was used and the best fitting model was determined from Akaike’s ‘An Information Criterion’. Adonis within the R package “vegan”^91^ was used for all PERMANOVA analyses. As there is no post-hoc^97^ test for Adonis, we used adjusted p values obtained from metagenomeHIT_zig in R through QIIME^83, 98^ to determine differentially abundant OTUs in treatments between life stages.

## Electronic supplementary material

Supplementary Information
