# Supplementary material for: Effects of contaminants of emerging concern on Megaselia scalaris (Lowe, Diptera: Phoridae) and its microbial community
Source: Sci Rep. 2017 Aug 15;7:8165. doi: 10.1038/s41598-017-08683-7 (PMC5557979; doi:10.1038/s41598-017-08683-7)

Effects of contaminants of emerging concern on *Megaselia scalaris* (Lowe, Diptera: Phoridae) and its microbial community

Marcus J. Pennington[ab*] ; Jason A. Rothman[ad]; Michael B. Jones[a]; Quinn S. McFrederick[ad]; Jay Gan[bc]; John T. Trumble[ab]

[a] Department of Entomology, University of California, Riverside, CA 92521, USA
[b] Graduate Program in Environmental Toxicology, University of California, Riverside, CA 92521, USA
[c] Department of Environmental Chemistry, University of California, Riverside, CA 92521, USA
[d] Graduate Program in Microbiology, University of California, Riverside, CA 92521, USA

* Corresponding Author: 417 Entomology Building, University of California, Riverside, CA, 92521, USA, mpenn001@ucr.edu

Running Head: *Megaselia scalaris* biology and microbial communities affected by CECs

Supp. Figure 1: Heatmap, after removing the family *Psedomonadaceae*, of the top 5% proportionately most abundant families by average OTUs of treat life-stage pairing. Increased red coloration is an increase in proportional abundance.

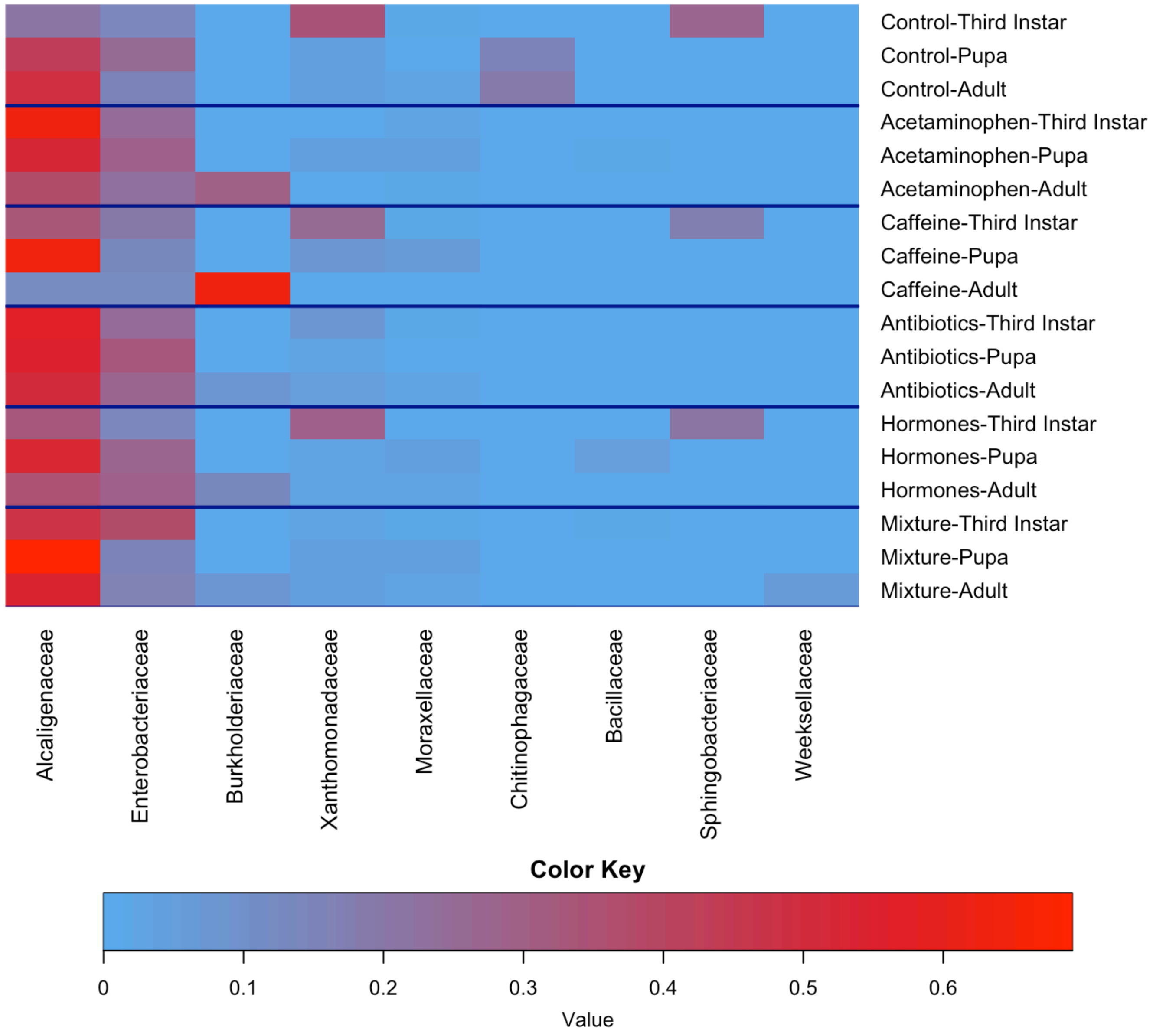

Supp. Figure 2: Non-metric multidimensional scaling plots of dissimilarities in treatments by life-stage.

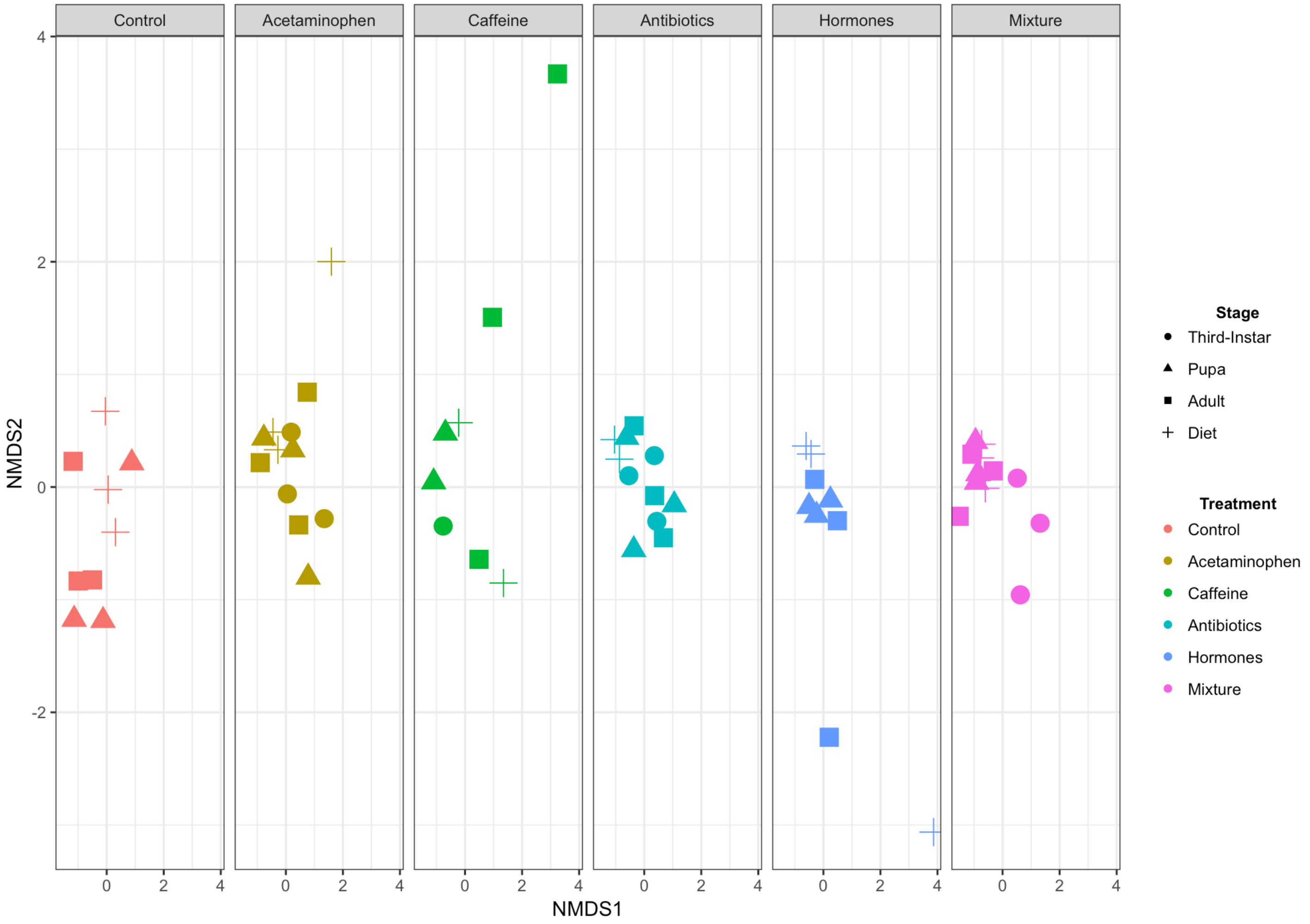

Supplement: Supplementary file 1 — Supplementary Information [file 41598_2017_8683_MOESM1_ESM.pdf]
